# Reinforcement Learning-Based Optimization of Ku-Band Low-Noise Amplifier

**DOI:** 10.3390/mi17050554

**Published:** 2026-04-30

**Authors:** Jiyong Chung, Hoyeon Shin, Seonho Shin, Yeonggi Kim, Saeed Zeinolabedinzadeh, Dongjin Ji, Ickhyun Song

**Affiliations:** 1 Department of Artificial Intelligence Semiconductor Engineering, Hanyang University, Seoul 04763, Republic of Korea; orangecounty@hanyang.ac.kr (J.C.); sin0514@hanyang.ac.kr (H.S.); a5731020@hanyang.ac.kr (S.S.); ygkim2460@hanyang.ac.kr (Y.K.); 2 Department of Electrical and Computer Engineering, University of Minnesota, Minneapolis, MN 55455, USA; 3 Department of Semiconductor Engineering, Seoul National University of Science and Technology, Seoul 01811, Republic of Korea; 4 Department of Electronic Engineering, Hanyang University, Seoul 04763, Republic of Korea

**Keywords:** GaN technology, low-noise amplifier (LNA), proximal policy optimization (PPO), reinforcement learning, transmission line

## Abstract

In this paper, we present a study on the automated design optimization of a wideband low-noise amplifier (LNA) operating in Ku-band (12 to 18 GHz) using proximal policy optimization (PPO), one of the widely applied reinforcement learning (RL) algorithms for engineering problems. As a target microwave active circuit, we select a two-stage LNA architecture, where transmission lines (TLs) are dominantly used for impedance matching and gain/noise optimization. For simplicity, all widths of TLs were fixed so that the characteristic impedance is 50 Ω, with lengths of TLs being set as design parameters. In addition, dimension variables of capacitors were treated as design parameters and, in total, we optimized 29 parameters. For target specifications, we set both $S_{11}$ and $S_{22}$ to be below −10 dB over the 12–18 GHz band and the noise figure (NF) to be below 2 dB. A total of 20,140 simulations were performed for training and the overall process took about 24 h. The results show that both the reward and the loss converged appropriately, achieving the target specifications successfully. For the final results, we performed up to 25 predictions, and the prediction process was terminated early if a solution meeting all target specifications was found within the given number of attempts. The device model used was a commercial 150 nm GaN high-electron-mobility transistor (HEMT) process technology.

## 1. Introduction

Designing radio-frequency (RF) or microwave circuits such as LNAs, mixers, and power amplifiers require substantial expertise, and even for experienced designers it is very challenging to optimize performance while simultaneously considering all the trade-offs among specifications such as gain, stability, chip area, gain flatness, input/output matching, noise figure (NF), and linearity. To achieve these multi-objective targets, designers iteratively tune the circuit over a long period of time [1,2,3]. Moreover, even if a circuit is designed to satisfy all of these conditions, it is difficult to know whether the resulting design has an optimized set of parameters. In addition, as modern microwave receivers increasingly demand operation at higher and wider frequency ranges, the design difficulty has become greater. To help reduce this extensive design time, some circuit simulators provide built-in optimization features that use methods such as genetic algorithms, the Newton method, and gradient descent to assist circuit optimization. These optimization algorithms, however, are useful for fine-tuning after a circuit has already been designed to be reasonably close to the target specifications. Thus, limitations for practical use exist in that they typically cannot perform an end-to-end optimization that satisfies all multi-objective specifications from scratch.

To overcome these challenges, recent efforts have continued to use artificial intelligence (AI) to shorten iterative parameter tuning of microwave circuits. Broadly, there are two representative AI-based approaches to circuit design. One approach uses AI to generate new circuit topologies—potentially discovering novel structures beyond those known to human designers. Studies about this approach explore topology generation by leveraging graph neural networks (GNNs) or large language models (LLMs) together with circuit netlists to synthesize novel circuit structures [4,5,6,7]. The other approach assumes a predefined topology and uses AI to determine the optimal device parameter values within that structure. Even in this latter research area, there remain many aspects that need further improvement and refinements. In other words, even if a new topology is generated, evaluating whether it is truly meaningful and practical still requires optimizing its device parameters using the second approach. In this regard, we focus on the latter approach, i.e., automated device-parameter optimization for a fixed, predefined circuit topology.

In the second approach, efficient parameter optimization is the key. As a representative gradient-free baseline, genetic algorithms (GAs) have been widely used for circuit optimization [8,9,10,11,12]. GAs are attractive for circuit optimization because they are gradient-free and can treat the simulator as a black box, which makes them robust to non-differentiable or noisy response and well suited to highly nonconvex, multimodal microwave design spaces; they also handle multi-objective trade-offs flexibly (e.g., via penalties or pareto optimization) and can naturally optimize mixed continuous and discrete variables, while being amenable to parallel evaluation of populations. On the other hand, these benefits come with practical drawbacks: GAs typically require a large number of simulations (high computational cost), can be inefficient for fine-tuning near an optimum compared with local or gradient-based methods, and their performance is sensitive to hyperparameters and constraint-penalty design, often leading to premature convergence or run-to-run variability.

Another approach is particle swarm optimization (PSO). PSO is a widely used optimization algorithm for circuits [13,14,15,16] and is inspired by the flocking behavior of migratory birds [17,18]. Similar to a group of birds flying to search for food, multiple simulator agents perform simulations while sweeping parameters toward better solutions. Because the agents explore not only individually but also by moving toward the swarm’s collective best (or global) tendency, while partially preserving each agent’s own direction, PSO has the advantage of being less likely to get trapped in local optima. Since many agents explore in parallel, however, PSO typically incurs high computational cost and requires long runtime due to the large number of simulations.

To overcome the limitations of the aforementioned algorithms, this paper adopts proximal policy optimization (PPO), a reinforcement learning-based optimization method. PPO is a policy-gradient algorithm that stabilizes training by constraining policy updates through a clipping mechanism, preventing the policy from changing too drastically in a single step [19,20,21]. This is important because policy-gradient methods can suffer from sudden performance collapse when updates become excessively large, and PPO mitigates this issue via clipping. In microwave circuit optimization, once a design reaches a reasonable performance level, further progress often depends on fine-tuning rather than broad random exploration. By learning how parameter updates affect the reward, PPO can capture effective fine-tuning patterns, reduce unnecessary trial-and-error, and accelerate convergence in subsequent episodes. In contrast, GA and PSO typically require evaluating many candidates per iteration, which leads to significantly higher simulation cost and longer runtime. From a simulation-efficiency perspective, PPO can therefore be more advantageous than GA and PSO. Moreover, many circuit specifications exhibit inherent trade-offs, and these relationships can be naturally incorporated into the reward function so that the agent learns to balance competing objectives during optimization. For these reasons, we employ PPO to optimize the circuit in this work.

This paper presents a PPO-based reinforcement learning framework for automated microwave circuit optimization under multi-objective specifications and expensive simulation constraints. We demonstrate the proposed approach on a Ku-band two-stage LNA implemented with transmission line network and parameterized passive components, resulting in a 27-dimensional design space. The experimental results show that the PPO agent can reliably learn effective fine-tuning strategies and achieve the target specifications with a practical number of simulations. The remainder of this paper is organized as follows: Section 2 describes the target circuit and optimization setup, Section 3 details the PPO formulation and reward design, Section 4 presents the simulation results, and Section 5 concludes the paper.

## 2. Methodology

### 2.1. LNA Circuit Topology

**Figure 1:**
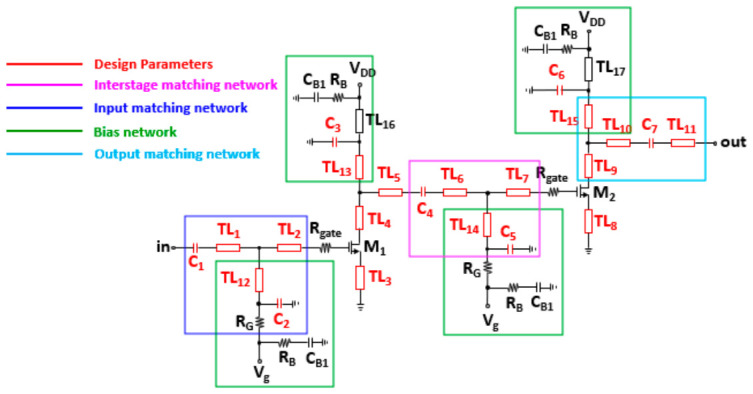
Schematic of the target two-stage Ku-band LNA. The elements highlighted in red indicate the design parameters optimized in this work.

In this section, we describe the target circuit to be optimized in this work and provide details of the PPO-based optimization approach. Figure 1 shows the schematic of the circuit optimized in this paper. The circuit is a two-stage distributed LNA designed using a commercial 150 nm GaN HEMT process. The gate bias voltage was fixed at −1.2 V and the supply voltage was fixed at 12 V, because for GaN device models the optimal operating point is pre-fixed and deviations may result in unreliable response in circuit behavior in performance prediction. In a distributed architecture, multiple stages are cascaded, and each stage is designed to provide a similar level of signal gain around the center frequency [22,23,24,25]. As a result, the overall amplifier exhibits a relatively uniform gain over a wide frequency range, which is a conventional approach for microwave LNA design.

For clarity, the circuit can be divided into an input/output matching network and a bias network. The bias network provides the required DC bias while preventing the high-frequency input signal from leaking into the DC supply path. All bias networks share a similar structure, except that an additional gate resistor, R_G_, is included only in the gate bias network. R_G_ helps protect the LNA from breakdown by limiting excessive DC current flowing into the gate. In addition, R_gate_ was set to a fixed value of 4 Ω as a resistor for circuit stability. C_B1_ and parallel capacitors (C_2_, C_3_, C_5_, C_6_) provide an AC ground over a wide frequency range, which improves the stability of the LNA. TL_12_, TL_13_, TL_14_, and TL_15_ function both as part of the matching network and as inductive elements, and they also prevent the high-frequency signal from leaking into the DC bias path.

The input and output matching networks are designed with the same topology. The input matching network consists of C_1_, C_2_, TL_1_, TL_2_, and TL_12_. TL_1_ and C_2_ are mainly used to achieve impedance matching at high frequencies, whereas C_1_ and TL_12_ are primarily used for matching at low frequencies. TL_3_ and TL_8_ implement inductive degeneration using transmission lines and are included to achieve power matching and noise matching simultaneously. In addition, C_1_ and C_3_ form part of the matching network while also serving as DC-blocking capacitors.

### 2.2. Problem Formulation as a Markov Decision Process

The circuit optimization problem is formulated as a Markov decision process (MDP) as follows:

1. State space *S_t_*: A vector at time t consisting of the penalty score computed from the target specifications, the normalized device parameter values, and the ratio of the episode termination step count to the maximum number of steps.
2. Action space *A_t_*: The action space is limited to continuous values between −1 and 1, and when the agent takes an action, that is, when it updates the device parameter values, the action indicates both the direction and the magnitude of the change.
3. Transition Probability: This indicates the likelihood of moving between states based on actions. This is defined by the following equation.

(1) $$P\left(S_{t+1}|S_{t},A_{t}\right)$$

4. Reward *R_t_*: The reward provides feedback to the agent. Based on the observation, a penalty is imposed when the target specifications are not satisfied, whereas no penalty is given when they are satisfied. A positive reward is granted only when all target specifications are met. The reward function is defined as follows,

(2) $$v_{m}\left(f\right)=s_{m}\left(x_{m}\left(f\right)-T_{m}\right),\;{\;s}_{m}=\{\begin{array}{c} 1,\;\;\;for\;upper\;bound\;constraints\\ -1,\;for\;lower\;bound\;constraints \end{array}$$

(3) $$p_{m}=\frac{1}{\left|F\right|}\sum_{f\in F}max(v_{m}\left(f\right),\;0)$$

(4) $$r=-\sum_{m\in M}w_{m}p_{m}-w_{trade}p_{S11}p_{NF}+b_{meet}$$

where $m\in \left\{S_{11},S_{22},S_{21},\;NF\right\},\;v_{m}(f)$ denotes the violation of the corresponding target specification at frequency *f*, *w_m_* denotes the weight assigned to each individual specification, and *b_meet_* = 5 only when all target specifications are satisfied over the entire operation frequency band; otherwise, *b_meet_* = 0. If *b_meet_* is set too low, the PPO agent may not clearly distinguish a fully feasible solution from a nearly feasible one, which weakens the incentive to satisfy all target specifications simultaneously and may cause training to remain around marginally violating solutions. In contrast, if $b_{meet}$ is set too high, the reward can become overly dominated by this bonus term, making the learning signal sparser and high-variance. In that case, the agent may focus excessively on the binary event of meeting all specifications rather than on gradual performance improvement, which can reduce training stability and lead to suboptimal convergence. Therefore, $b_{meet}$ should be chosen so that it is large enough to encourage full specification satisfaction, but not so large that it overwhelms the penalty-based shaping terms. In an LNA, input matching and noise matching are in a trade-off relationship and cannot be simultaneously optimized without compromise. To reflect this relationship in the reward function, an additional weighted term based on the product of $p_{S11}$ and $p_{NF}$ was introduced.

The objective of the MDP can be expressed as maximizing the expected cumulative reward,

(5) $$\underset{\pi }{\max}E\left[\sum_{t=0}^{T}\gamma^{t}R_{t}\right]$$

where π denotes the policy, E denotes the expectation, $R_{t}$ is the reward at time step t, γ is the discount factor that balances immediate and future rewards, and T is the time horizon.

### 2.3. PPO Implementation Details

**Figure 2:**
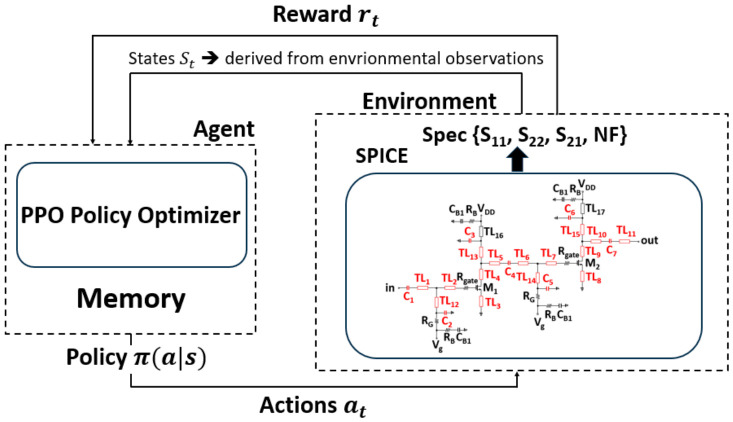
Block diagram of PPO-based circuit optimization framework.

Figure 2 shows the overall architecture of the PPO-based circuit optimization system. The agent learns by interacting with the environment. Starting from the initial parameter values, the agent takes an action in the environment, and the environment returns the corresponding reward to the agent through a simulation program with integrated circuit emphasis (SPICE) tool. Based on this reward, the agent learns in which direction and by how much the parameters should be adjusted in the next step, and updates its policy accordingly. By repeating this process, the agent gradually learns a more effective parameter-tuning policy.

In Table 1, the main PPO training hyper parameters used in this work are summarized. The hyperparameters were selected based on previous studies that applied PPO and were adjusted to suit the circuit optimization environment considered in this work [19,26]. A multi-layer perceptron (MLP) policy was adopted, and the rollout length was set to 265, meaning that 265 environment interactions were collected before each policy update. It was chosen empirically so that the PPO agent could gather enough samples, approximately ten episodes, before each policy update. The batch size was also set to 265 so that the entire rollout was used for each update. The learning rate was set to $3\times 10^{-4}$, while the discount factor γ and the GAE parameter λ were set to 0.99 and 0.95, respectively, in order to stably account for both immediate and future rewards. In addition, the PPO clipping range was set to 0.2 to prevent excessively large policy updates, and the maximum gradient norm was limited to 0.5 to improve training stability. The action scale was set to 0.08 so that the agent could adjust the circuit parameters gradually within the predefined feasible ranges. Since a microwave LNA is highly sensitive even to small variations in its device parameters, such gradual updates are beneficial because they allow the agent to explore the design space effectively while still escaping unfavorable initial conditions. Finally, the maximum number of steps per episode was set to 25.

A total of 29 device parameters were selected as optimization variables, including 11 general transmission-line lengths (from TL_1_ to TL_11_), 4 bias transmission-line lengths (from TL_12_ to TL_15_), and width and length of 7 capacitors (from C_1_ to C_7_). The lengths of the general transmission lines were initialized to 200 μm and optimized within the range of 9 μm to 500 μm. The lengths of the bias transmission lines were initialized to 1000 μm and optimized within the range of 700 μm to 2000 μm. The width and length of the capacitors were both initialized to 50 μm and optimized within the range of 16 μm to 200 μm. These parameters were chosen because they have a direct influence on the matching characteristics, gain response, and overall wideband performance of the circuit. For all variables, lower and upper bounds were predefined based on practical design feasibility and process constraints, and the PPO agent was allowed to adjust the parameters only within these ranges. This bounded parameter setting not only ensured physically realizable designs but also improved the stability and efficiency of the optimization process.

## 3. Results

In this section, the training behavior and final optimization results of the proposed PPO-based framework are presented. First, the learning performance of the agent is analyzed using the reward, training value loss and episode-length trends observed during training. Then, the LNA performance of the optimized circuit is evaluated in terms of the target specifications over the desired frequency band. Through these results, the effectiveness of the proposed method for automated circuit optimization is verified.

**Figure 3:**
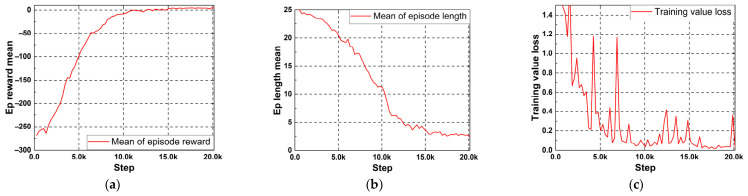
(**a**) Episode reward mean. (**b**) Mean of episode length. (**c**) Training value loss. Here, “step” denotes an individual simulator call, i.e., one action taken by the agent followed by one circuit simulation and reward evaluation.

As shown in Figure 3, the PPO agent exhibited a stable learning trend throughout training. Here, one step on the horizontal axis denotes one circuit simulation run. In Figure 3a, the mean episode reward increased steadily from a large negative value and gradually converged to a value close to zero, indicating that the agent progressively reduced the specification violations and approached the target design goals. At the same time, the mean episode length decreased from nearly the maximum allowed number of steps to approximately 2–3 steps, implying that the agent became increasingly efficient at finding parameter updates that satisfied the target specifications with fewer interactions with the environment [See Figure 3b]. Considering the complexity of a 29-dimensional action space, the proposed optimization is formulated as a constrained local refinement around a sensible initial design rather than an unconstrained global search. Therefore, rapid improvement within the first few steps is plausible in this setting. Importantly, convergence is not inferred solely from the early discovery of high-performing solutions, but also from the fact that continued training yielded only marginal additional improvement, indicating practical saturation under the current search setting. These results suggest that the proposed PPO-based framework successfully learned an effective parameter-tuning policy for the circuit optimization task.

The training value loss in Figure 3c also showed an overall decreasing trend, despite several fluctuations during the early and intermediate stages of training. Such fluctuations are common in reinforcement learning because the value function is updated from continuously changing policy-generated data. Nevertheless, the value loss gradually stabilized at a low level as training progressed, which indicates that the value network learned to predict the long-term return more accurately. Taken together, the trends in episode reward, episode length, and value loss demonstrate that the PPO agent achieved stable convergence and improved optimization efficiency over the course of training.

**Figure 4:**
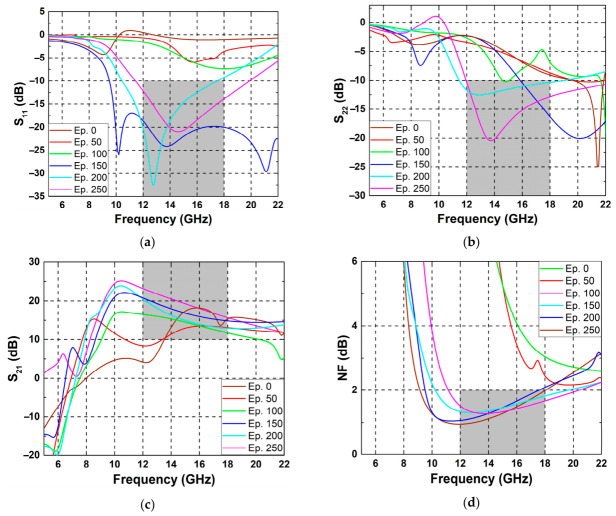
(**a**) S_11_, (**b**) S_22_, (**c**) S_21_, (**d**) NF results from different episodes during training. The gray region indicates the target specification range.

Figure 4 shows the simulation results at the end of selected episodes from the later stage of training. Specifically, the results were plotted every 50 episodes after episode 2000. As can be seen from the S_11_ and S_22_ response [See Figure 4a and Figure 4b, respectively], reasonable matching was achieved below −10 dB over the operating frequency range from 12 to 18 GHz. The S_21_ response shown in Figure 4c also remained above 10 dB throughout the target band. Likewise, the NF response in Figure 4d stayed below the target value of 2 dB over the operating frequency range.

In overall LNA performance, some issues need to be addressed: when the LNA is not properly optimized for stability, abnormal behaviors in impedance matching can happen. As shown in Figure 4a,b, S_11_ or S_22_ may go above 0 dB, which implies unstable operation or potential oscillation. Therefore, it is necessary to use the stability factor (K-factor) for validating stable behavior of the LNA. Next, while the gain (S_21_) remains above the target value over the operating band, its peak is not around the center frequency of Ku-band. In addition, the fluctuations in gain within the operation band are not negligible. This indicates that the amplifier does not yet exhibit fully adequate for wideband operation. This limitation may be improved by introducing additional constraints into the reward function during training.

**Figure 5:**
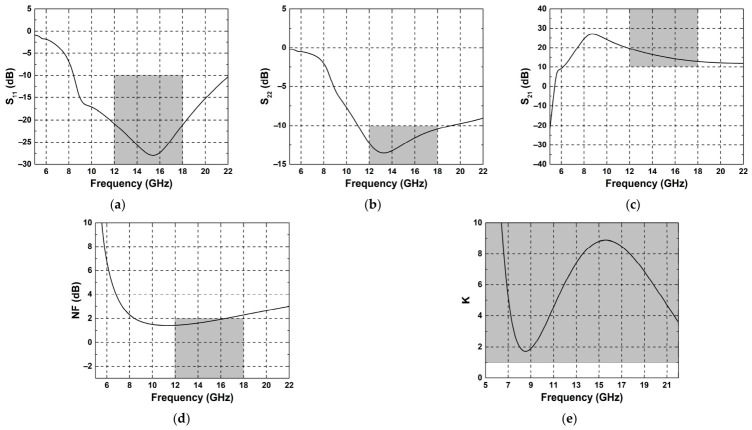
(**a**) S_11_, (**b**) S_22_, (**c**) S_21_, (**d**) NF, (**e**) K-factor predicted by trained model. The gray region indicates the ranges of target specifications.

Using the trained model obtained through the above process, a final prediction was performed. Figure 5 shows the simulation results obtained using the final predicted device parameters. S_11_, S_22_, S_21_, NF, and K-factor versus frequency are shown in Figure 5a, Figure 5b, Figure 5c, Figure 5d, and Figure 5e, respectively. Most target specifications were satisfied over the operating frequency band. For the final prediction, a total of 10 episodes were carried out. At the end of each episode, the parameter set with the highest reward was selected, and the best parameter sets from all episodes were then compared in terms of reward. The parameter set with the highest overall reward was chosen as the final prediction. Table 2 presents the corresponding parameter values.

As shown in Table 3, the proposed LNA does not provide a clear advantage over previously reported works in all circuit-level performance metrics. In particular, its gain is moderate and its dc power consumption is relatively high [27]. The relatively high dc power consumption is attributable not only to the two-stage topology and fixed bias condition, but also to the fact that power consumption was not explicitly optimized in this work. In addition, the transistor size was fixed, so the trade-off between RF performance and power efficiency was not fully explored.

**Figure 6:**
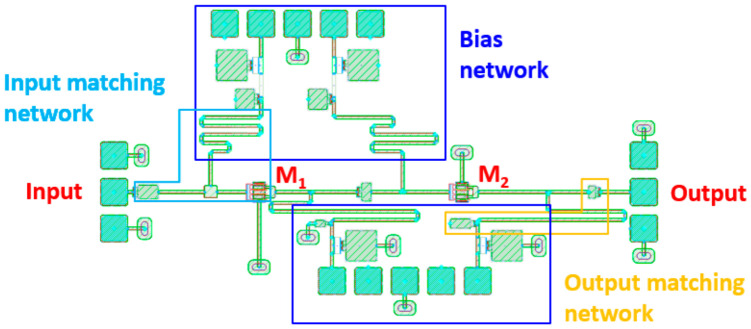
The layout of the performance-optimized Ku-band LNA.

Nevertheless, the circuit still exhibits reasonable LNA performance, including operation over the 12–18 GHz band, input/output return loss of 20.8/10.5 dB, and a noise figure of 1.45–2.3 dB. These results indicate that the optimized design can operate as a practically acceptable wideband LNA. Therefore, the significance of this work lies primarily in demonstrating the feasibility of PPO-based automated microwave circuit optimization rather than achieving the best absolute circuit performance. Unlike the conventional designs that are manually optimized by experienced designers, the proposed approach automatically learned a device-parameter tuning policy and successfully found parameter sets satisfying the target specifications over the 12–18 GHz band. In addition, Figure 6 presents the final chip layout of the optimized LNA with the given design parameters, which occupies an area of 2.88 mm^2^ (1.26 mm × 2.30 mm). Most of the chip area is composed of multi-stage configurations, TLs, and pad structures. For long TLs with straight length, they were manually bent to form a meander line for generating physically realizable layout. This result confirms that the PPO-optimized design can be regarded as a proof-of-concept. Therefore, reinforcement learning can be effectively applied to practical Ku-band wideband LNA design, whereas there is still considerable room for improvement in terms of circuit performance, area efficiency, and reward design.

## 4. Discussion

The results of this work demonstrate that PPO can serve as an effective optimization framework for RF circuit design under a fixed topology. By interacting with a circuit simulator environment, the agent learned how to adjust device parameters so as to satisfy multiple design specifications over the target frequency band. In particular, the proposed method was able to achieve the desired matching, gain, and NF targets in the final prediction, showing that reinforcement learning can be applied not only to abstract benchmark problems but also to practical RF circuit optimization tasks with physically meaningful design variables.

At the same time, the training results also reveal several important limitations and future research directions. Although the agent generally learned parameter sets that satisfied the target specifications over the 12–18 GHz band, the intermediate results showed that some performance metrics were still not fully controlled outside the target band. For example, the NF exhibited an undesirable rise in the low-frequency region, which is presumed to be related to the fact that the stability metric was not explicitly considered in the reward design. In addition, while the gain remained above the target value, sufficient gain flatness was not consistently obtained across the operating band. These observations indicate that the final circuit behavior is strongly influenced by how the reward function is formulated, and that additional design objectives or penalty terms may be required to guide the agent toward more practically desirable solutions.

Another important point is that the proposed method optimized only device parameters within a predefined circuit topology. This means that the effectiveness of the overall design still depends on the suitability of the initial circuit structure. In other words, PPO can successfully improve a given topology, but it does not guarantee that the chosen topology itself is globally optimal. Nevertheless, this limitation does not reduce the value of the proposed approach, because practical RF design often starts from a conventional and physically validated circuit structure, after which parameter optimization becomes the core challenge. In this context, the proposed PPO-based framework provides a meaningful and scalable alternative to conventional heuristic optimization methods such as GA and PSO. For example, as reported in [8], the learning curves of GA- and PSO-based mixer optimization show that a large number of simulations are required before noticeable performance improvement is obtained. This suggests that such methods may require substantial simulation cost to reach the target performance, although a direct quantitative comparison under the same design condition remains an important topic for future work.

Furthermore, this line of research may be extended toward topology generation by using graph neural networks (GNNs). If the pins of each device are represented as vertices and the electrical connections between pins of different devices are represented as edges, a circuit can be modeled as a graph structure. Based on such a representation, GNN-based methods may be able to explore and generate new circuit topologies beyond predefined structures. After identifying a new topology using a GNN, the PPO-based parameter optimization framework proposed in this work could then be applied to optimize that topology. In this sense, the present study may contribute to a broader AI-based microwave circuit design flow that combines topology generation and parameter optimization.

From a broader perspective, the results suggest that reinforcement learning has strong potential for automated RF circuit design, especially in problems involving expensive simulations, multiple trade-offs, and high-dimensional parameter spaces. In particular, the proposed PPO-based framework can reduce repetitive manual tuning effort by automatically learning how to update circuit parameters toward multi-objective target specifications through repeated interaction with the circuit simulator. In this sense, the method can serve as a practical design-assistance tool for simulation-driven microwave circuit optimization. However, further improvements are still needed for wider practical adoption. These include more sample-efficient training, more refined reward design, layout-award optimization, incorporation of additional performance constraints such as gain flatness and stability, and validation on more complex microwave blocks beyond the two-stage LNA considered in this work.

A particularly important direction for future work is layout-aware optimization, since in practical microwave circuit design the transmission-line length is closely related to both RF loss and broadband matching characteristics. Therefore, future research will investigate an optimization framework that explicitly considers the trade-off among transmission-line length, loss, and broadband matching performance during the design process.

## 5. Summary

In this work, a PPO-based reinforcement learning framework was proposed for the automated optimization of a Ku-band LNA. A two-stage distributed LNA operating in a 12–18 GHz band was selected as the target circuit, and a total of 29 design variables, including TL lengths and capacitor dimensions, were optimized within a fixed topology. By integrating a circuit simulator with a Python-based execution environment (version 3.12.2), the PPO agent iteratively adjusted the device parameters and learned a parameter-tuning policy from simulation-based rewards. The training results showed that the proposed framework achieved stable learning behavior, as confirmed by the increase in episode reward, the decrease in episode length, and the stabilization of value loss during training.

The final predicted design satisfied the target specifications over the operating frequency band, demonstrating that PPO can effectively handle multi-objective microwave circuit optimization under a predefined topology. In addition, the final chip layout confirmed that the optimized design is physically realizable. While the overall circuit performance numbers are not yet superior to those of previously reported high-frequency LNAs, the significance of this work lies in verifying that a reinforcement learning algorithm can autonomously optimize a practical microwave circuit with physically meaningful design parameters. Hence, this work provides a proof-of-concept study for AI-assisted microwave circuit design. Future work will focus on improving the reward function by incorporating additional constraints such as gain flatness and stability, reducing chip area and power consumption, and extending the framework to more complex circuit topologies.

## Figures and Tables

**Table 1:** List of Hyperparameters and Their Values for PPO.

Hyperparameters	Value *
Maximum episode steps	25
Action scale	0.08
Batch size [samples]	265
Rollout length [steps]	265
Learning rate	3 × 10^−4^
Discount factor, γ	0.99
GAE parameter, λ	0.95
Clipping range for policy updates	0.2
Entropy coefficient	0
Value loss coefficient	0.5
Maximum gradient norm	0.5
Total time steps	20,140

* All values are unitless unless otherwise specified.

**Table 2:** Device Parameters Predicted by PPO.

Device Parameters	Value (μm)	Device Parameters	Value (μm)	Device Parameters	Value (μm)
Length of TL_1_	189.6	Length of TL_9_	278.56	Width/Length of C_2_	79.4/71.4
Length of TL_2_	121.4	Length of TL_10_	158.39	Width/Length of C_3_	21.9/37.6
Length of TL_3_	268.2	Length of TL_11_	121.4	Width/Length of C_4_	69.8/33.7
Length of TL_4_	137.2	Length of TL_12_	1208	Width/Length of C_5_	79.4/72.0
Length of TL_5_	182.1	Length of TL_13_	1208	Width/Length of C_6_	33.7/79.4
Length of TL_6_	121.4	Length of TL_14_	1128.9	Width/Length of C_7_	46.2/24.3
Length of TL_7_	186.1	Length of TL_15_	981.4		
Length of TL_8_	121.44	Width/Length of C_1_	50.9/78.4		

**Table 3:** Performance Comparison with Previously Reported Microwave LNAs.

Ref.	Process	Freq. (GHz)	Gain (dB)	I/O RL (dB)	NF (dB)	P_DC_ (mW)
[22]	0.15 μm GaN-Si	25–31	≥21	≥12/10	2.4–2.9	300
[23]	0.1 μm GaN-HEMT	18–31	21	≥10/10	0.8–1.2	410
[24]	0.1 μm GaN/Si-HEMT	18–56	16–21.5	≥8/8	2.2–4.4	1400
[25]	0.25 μm GaN	6–18	25.46	≥10.5/12	2–2.7	490
[28]	0.15 μm GaN-HEMT	10.7–12.7	21.9–24.5	≥15.4/15	1.7–2.1	217
[29]	90 nm T-gate GaN	18–44	14.3–24.4	≥10/10	1.5–2.5	630
[30]	0.15 μm AlGaN/GaN HEMT	8–11	>16.8	>9.8/10	<1.7	135
[31]	0.15 μm GaN-HEMT	8–12	>9.4	≥10/8.4	<1.9	270
This work	0.15 μm GaN-HEMT	12–18	>10	≥20.8/10.5	1.45–2.3	1192

## Data Availability

The original contributions presented in this study are included in the article. Further inquiries can be directed to the corresponding author.
